# Continuous Glucose Monitoring Systems: A Review

**DOI:** 10.3390/diagnostics3040385

**Published:** 2013-10-29

**Authors:** Sandeep Kumar Vashist

**Affiliations:** HSG-IMIT—Institut für Mikro-und Informationstechnik, Georges-Koehler-Allee 103, 79100, Freiburg, Germany; E-Mail: sandeep.kumar.vashist@hsg-imit.de; Tel.: +49-761-203-7252; Fax: +49-761-203-73299

**Keywords:** continuous glucose monitoring systems, diabetes management, invasive, non-invasive

## Abstract

There have been continuous advances in the field of glucose monitoring during the last four decades, which have led to the development of highly evolved blood glucose meters, non-invasive glucose monitoring (NGM) devices and continuous glucose monitoring systems (CGMS). Glucose monitoring is an integral part of diabetes management, and the maintenance of physiological blood glucose concentration is the only way for a diabetic to avoid life-threatening diabetic complications. CGMS have led to tremendous improvements in diabetic management, as shown by the significant lowering of glycated hemoglobin (HbA1c) in adults with type I diabetes. Most of the CGMS have been minimally-invasive, although the more recent ones are based on NGM techniques. This manuscript reviews the advances in CGMS for diabetes management along with the future prospects and the challenges involved.

## 1. Introduction

Diabetes is increasing worldwide at an unprecedented pace and has become a serious health concern during the last two decades. It is a major cause of mortality in the age group of 20–79 years. Based on its rapidly increasing incidence, it has been declared a global epidemic by the World Health Organization (WHO). The annual cost associated with diabetes management, *i.e.*, US $376 billion, will increase to US $490 billion in 2030 [1]. In 2004, WHO estimated the number of diabetics to increase from 171 million in 2000 to 366 million by 2030 [2]. However, the current estimates by International Diabetes Federation states 372 million diabetics in 2012 itself and predicts 552 million diabetics by 2030 [3,4,5]. The disease state characterized by persistently high glucose levels is referred to as diabetes mellitus. Type I diabetes mellitus refers to the juvenile offset stage when the pancreas cannot produce sufficient insulin, while type II diabetes mellitus reflects the inability of the body to use the secreted insulin.

**Figure 1:**
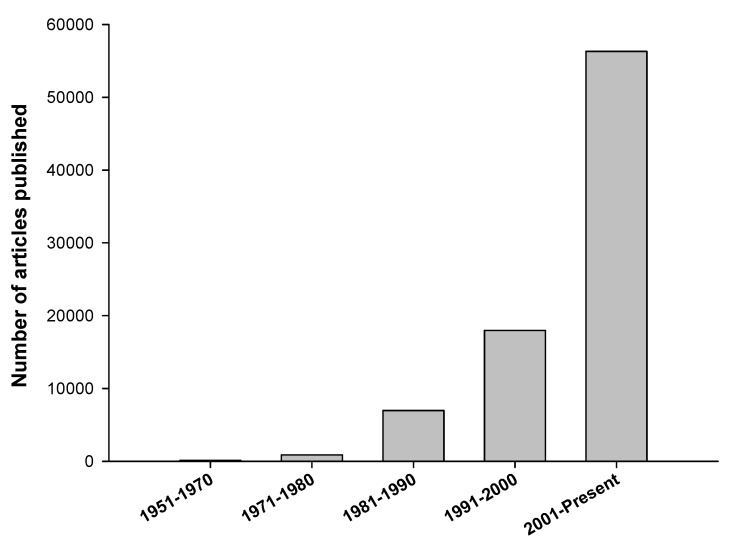
The number of articles published on glucose monitoring during the mentioned period. Data was taken on July 1, 2013, from Thomson Reuter’s Web of Knowledge (New York, NY, USA) using “glucose monitoring” in the timespan.

There have been continuously increasing research efforts in the field of glucose monitoring during the last few decades (Figure 1). The frequent monitoring of blood glucose is critical for diabetic management, as the maintenance of physiological glucose level, *i.e.*, 4–8 mM (72–144 mg/dL), is the only way that a diabetic can lead a healthy lifestyle by avoiding life-threatening diabetic complications, such as diabetic retinopathy, kidney damage, heart diseases, stroke, neuropathy and birth defects [6]. The Action to Control Cardiovascular Risk in Diabetes (ACCORD) trial demonstrated that the lowering of blood glucose in patients with high glycated hemoglobin (HbA1c) increased the long-term mortality significantly, due to more episodes of severe hypoglycemia [7,8,9] and the persistently higher HbA1c levels [10]. The Diabetes Control and Complications Trial [11] showed a ~60% decrease in the development of diabetic retinopathy and nephropathy in type 1 and type 2 diabetics by intensive glycemic control. Similarly, the UK Prospective Diabetes Study [11] showed a 25% decrease in overall microvascular complication rate in type 2 diabetics by intensive glycemic control. All the diabetic complications are most likely due to the Maillard reaction [12] of glucose via Schiff’s base products and subsequent stable glycation reactions with the amino terminals of proteins or nucleic acids. The heterogeneous products, named Amadori products, chemically constitute keto/amino derivatives, such as HbA1c [13]. Such advanced glycation end-products (AGEs) [14,15] are highly stable and are only degraded by macrophages. The excessive deposition of AGEs in diabetics [16,17,18] plays a pivotal role in the development of diabetic complications [19,20,21,22,23,24,25,26]. Moreover, they alter the structure and function of biomolecules and increase oxidative stress [27]. They represent mediators of “glycemic memory”, which is responsible for the long-lasting detrimental effects on the vasculature in different organs, intima media thickening and contributes to the pathogenesis of several diabetic complications, such as nephropathy, neuropathy, retinopathy, cardiovascular diseases and atherosclerosis [28,29]. The formation of AGEs directly correlates with glycemic control. Therefore, continuous glucose monitoring (CGM) is of the uttermost importance, as it can control the formation of AGEs, which leads to a reduction of the HbA1c level [30]. The American Diabetes Association (ADA) recommends an HbA1c level of <7% for most adults with diabetes [31]. Low and high HbA1c values have been shown to be associated with increased all-cause mortality and cardiac events [32]. Presently, the determination of HbA1c is an established procedure to evaluate long-term glycemic control and the quality of care provided to diabetics [33].

The most widely used glucose monitoring devices are blood glucose meters [34] based on minimally-invasive fingerstick tests that have a substantial market worth US $6.1 billion [35], which is majorly dominated by Roche Diagnostics, Bayer, Abbott, Medtronic and LifeScan. However, the recent trend is shifting towards non-invasive glucose monitoring (NGM) [36], as it alleviates the pain and suffering of diabetics, who have to frequently prick their skin, more than four times a day, for blood glucose measurements. The most extensively used NGM techniques are Raman spectroscopy and absorbance spectroscopy, although a wide range of other techniques have also been demonstrated. NGM obviates the need of a strip that makes the repeated measurements highly cost-effective, but it lacks the precision and specificity of blood glucose meters.

Blood glucose meters can effectively measure glucose, even at low levels. They enable the achievement of an optimal glucose level of 5–7.2 mmol/L for less than 30% of the day [37]. However, several continuous glucose monitoring systems (CGMS), which are presently more than five years old, can also detect glucose at low levels [38]. There is an immense need for CGMS [39,40,41,42,43,44,45], as it provides healthcare professionals and patients with highly useful information pertaining to the trends in glucose concentration throughout the day. This enables the prevention of hypoglycemic episodes and enables the diabetic to maintain the physiological glucose concentration level, *i.e.*, euglycemia with reduced glycemic variability. The advantages of CGM in diabetic management are very clear to the diabetic community, as it lowers the HbA1c level [46,47,48,49,50]. The Diabetes Control and Complications Trial had previously demonstrated the long-term benefits of lowering the HbA1c levels [51]. CGMS provide a large number of glucose measurements, which are highly useful to healthcare professionals to determine the fasting and postprandial blood glucose levels for better adjustment of the insulin dose, the effect of exercise or physical activity on glucose levels and the detection of unrecognized hypo- or hyper-glycemia. A prospective study demonstrated that CGMS reduced the risk of nocturnal hypoglycemias in 75% of type 1 diabetics [52]. This enabled the identification of glycemic excursions and postprandial hyperglycemia and improved the metabolic changes in the therapeutics of type 1 diabetic patients [53,54]. The assessment of glycemic control in type 2 diabetics is usually done by monitoring HbA1c, fasting plasma glucose and postprandial glucose. However, the glucose variability is also equally important, as both upward and downward fluctuations of glucose level are potent activators of oxidative stress. CGMS enables the detection of the mean amplitude glucose excursions (MAGE) index, which is the gold standard for determining glucose variability [55]. 

**Figure 2:**
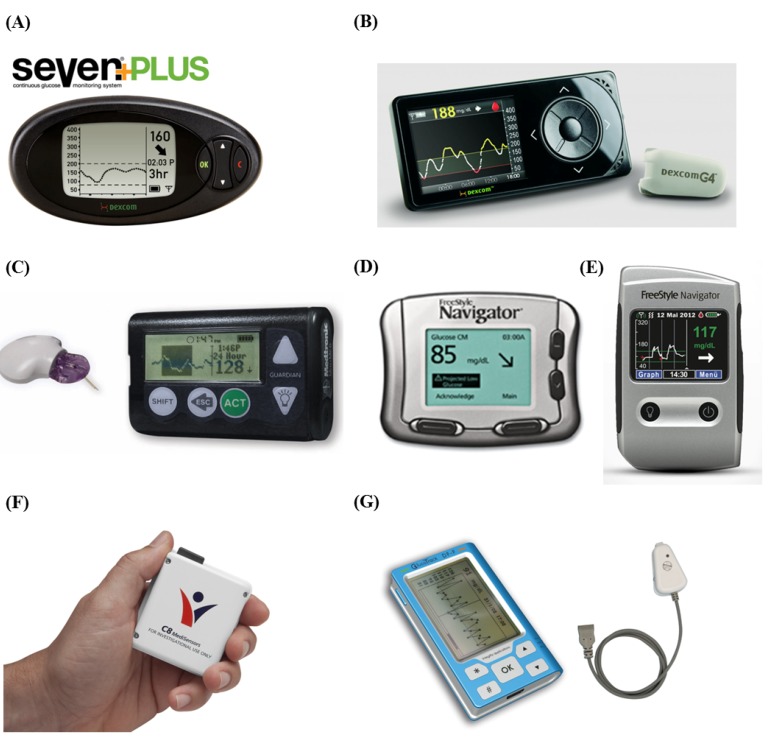
Continuous glucose monitoring systems (CGMS). (**A**) Dexcom SEVEN^®^ Plus, (**B**) Dexcom G4^TM^, (**C**) Guardian REAL-time, (**D**) FreeStyle Navigator^®^, (**E**) FreeStyle Navigator II, (**F**) HG1-c and (**G**) GlucoTrack^TM^.

Most of the CGMS employ minimally-invasive approaches that use subcutaneous sensors to determine the glucose concentration in interstitial fluid. Therefore, they cause discomfort to patients, require more frequent calibration by fingerstick tests and cannot be used for more than a few days, as the sensor is prone to biofouling. The main limitation is their extremely high cost, which is beyond the reach of most diabetics. This strictly limits their use to only selected clinical scenarios, where they are critically required. The most recent NGM technique-based CGMS by C8 Medisensors was a highly prospective development, due to its significantly reduced operational cost and pain-free monitoring procedure, which will stimulate diabetics to use CGMS for more effective diabetic management. This manuscript aims to provide a critical review of CGMS, taking into account the technology developments, the current state-of-the-art, the challenges involved and future prospects. The various CGMS developed to date are shown in Figure 2 and specified in Table 1. 

**Table 1:** Continuous glucose monitoring systems. MARD, mean absolute relative difference; EGA, error grid analysis; CE, Conformité Européenne; DG4P, Dexcom G4^TM^ PLATINUM; FN, FreeStyle Navigator^®^.

CGMS (Company)	Device Characteristics
Dexcom SEVEN^®^ Plus (Dexcom)	*Target site:* skin *Technology used:* glucose sensor; sensor lifespan: 168 h; length of sensor probe: 13 mm; gauge of sensor probe: 26 mm; sensor warm up: 2 h; calibration every 12 h, but twice within the first 30 min. *Advantages:* use FDA-approved glucose sensor that can be used for up to seven days; precisely detect hypoglycemic glucose level; water-resistant transmitter with built-in battery lasting one year; transmits glucose sensing data to the handheld receiver within five feet range every 5 min; receiver shows trends and current glucose level; stores up to 30 days of glucose trends along with a record of activities and events; comes with Dexcom*^®^* Data Manager 3 software, which provides better insight into the ways to improve glycemic control. *Disadvantages:* invasive; is a previous generation of CGMS that is not being used anymore; requires calibration with blood glucose testing; requires a change of the sensor after a few days. *Reliability:* MARD in euglycemia region = 18.4%; MARD in hypoglycemia region = 22.5%; Clarke EGA in euglycemia region = 98.3% in A + B, 91.3% in A; Clarke EGA in hypoglycemia region = NA [56]. MARD in hypoglycemia region = 21.5% [57]; aggregate MARD = 16% [58]; 76% of DG4P sensors had an individual MARD of ≤20%; DG4P had a mean absolute difference of 16 mg/dL for hypoglycemia (Yellow Springs Instrument Company’s YSI blood glucose analyzer < 70 mg/dL) [58]. *Cost:* US $1,158 for CGMS and US $349 for four sensors. *Availability:* USA, UK, European Union, Asian and Latin American countries that recognize the CE mark.
Dexcom G4^TM^ PLATINUM (Dexcom)	*Target site:* skin *Technology used:* glucose sensor; sensor lifespan: 168 h; length of sensor probe: 13 mm; gauge of sensor probe: 26 mm; sensor warm up: 2 h; calibration every 12 h, but twice within the first 30 min. *Advantages:* compact, wearable and light-weight; measures glucose continuously every 5 min; transmit glucose readings to the receiver up to a distance of 6 m; employs a very thin glucose sensor that can be used for up to seven days and has the same diameter as a human hair; high precision; receiver with colored screen and alarm alerts for high and low glucose levels and when the glucose levels are rising or falling quickly; comes with Dexcom STUDIO^TM^ data management software, which enables simple navigation and an illustrative visual for the quick and easy identification of glucose patterns. *Disadvantages:* invasive; requires calibration with blood glucose testing every 12 h; requires a change of the sensor after a few days. *Reliability:* aggregate MARD = 13%; 90% of DG4P sensors had an individual MARD of ≤20%; DG4P had a mean absolute difference of 11 mg/dL for hypoglycemia (YSI < 70 mg/dL) [58]. *Cost:* US $1,198 for CGMS and US $349 for a four-pack of sensors. Daily cost of US $6.70. *Availability:* USA, European Union, Asian and Latin American countries that recognize the CE mark.
Guardian^®^ REAL-time (Medtronic)	*Target site:* skin *Technology used:* glucose sensor; sensor lifespan: 72 h; length of sensor probe: 14 mm; gauge of sensor probe: 23 mm; sensor warm up: 2 h; calibration at 2 h, 8 h and then every 12 h. *Advantages:* measures glucose levels in the interstitial fluid every 10 s; wireless transmitter attached to a glucose sensor transmits average glucose reading to the monitor every 5 min; employs a glucose sensor that can be used for up to six days; displays glucose trends on the receiver; alarm alerts for high and low glucose levels; predictive alerts notify the user up to 30 min before they are predicted to reach a personal low or high glucose level; *Disadvantages:* invasive; requires calibration with blood glucose testing; requires a change of the sensor after a few days. *Reliability:* MARD in euglycemia region = 13.3%; MARD in hypoglycemia region = 13.8%; Clarke EGA in euglycemia region = 98.9% in A + B, 91.3% in A; Clarke EGA in hypoglycemia region = 84.4 in A + B, 81.9 in A [56]; 72% in A zone and 27.6% values in B zones of Clarke EGA; and a MARD of 17.6% [59]. *Cost:* US $1,400 for CGMS and US $50 for a sensor. *Availability:* USA, Europe, India
FreeStyle Navigator^®^ (Abbott)	*Target site:* skin *Technology used:* glucose sensor; sensor lifespan: 120 h; length of sensor probe: 6 mm; sensor warm up: 10 h; calibration at 10 h, 12 h, 24 h and 72 h. *Advantages:* measures glucose level every 1 min; employs a glucose sensor that can be used for CGM for up to five days; transmits glucose measurement data to the receiver up to 3 m; cellphone-sized receiver is a stand-alone unit with integrated blood glucose testing; built-in FreeStyle blood glucose meter provides convenient calibration using the test strips used in Abbott’s FreeStyle Lite blood glucose meter; receiver’s screen displays the current glucose level, glucose trend and the continuous glucose measurements; transmitter is compact, light, waterproof and battery-powered; reliable early-warning alarms alert for high and low glucose levels up to 30 min in advance. *Disadvantages:* invasive; requires calibration with a built-in blood glucose meter; requires a change of the sensor after a few days. *Reliability:* MARD in euglycemia region = 11.8%; MARD in hypoglycemia region = 7.4%; Clarke EGA in euglycemia region = 98.6% in A + B, 93.7% in A; Clarke EGA in hypoglycemia region = 97% in A + B, 95.5% in A [56]; MARD in hypoglycemia region = 29.8% [57]. 93.7%, 3.6% and 2.8% readings in the A, B and clinical error regions of EGA, respectively [60]; the clinical accuracy of the FN in the first 10 h and subsequent hours were 92.6% and 94.2%, respectively; FN has the MARD and median absolute relative difference of 12.8% and 9.3%, respectively [61]; the readings in the A and B zones of EGA were 81.7% and 6.7%, respectively. *Cost:* US $1,000 for the receiver and transmitter and US $375 for a pack of six sensors. *Availability:* USA, UK
FreeStyle Navigator II (Abbott)	*Target site:* skin *Technology used:* glucose sensor; sensor lifespan: 120 h; length of sensor probe: 6 mm; sensor warm up: 10 h; calibration at 10 h, 12 h, 24 h and 72 h. *Advantages:* compact, lightweight and comfortable to wear in comparison to its predecessor, *i.e.*, FreeStyle Navigator^®^; employs a glucose sensor that can be used for CGM for up to five days; compact transmitter with capsuled battery; compact and easy-to-use receiver with capsuled reloadable battery cell and illuminated color display; provides continuous glucose levels every minute, blood glucose averages of the last 10 min and the low and high blood glucose levels; provides greater flexibility, due to non-interference with the everyday activities of the diabetic; provides glucose trend and its direction; provides early warning alarm alerts if there is the risk of too low or too high glucose levels; works during showering, swimming, exercising or traveling and can transmit glucose sensing data to the receiver at an extended range of 30 m; can also be used for children above six years of age and adolescents under the supervision of an adult; built-in FreeStyle blood glucose meter; appropriate for evaluating the blood glucose fluctuations in the short term and additionally provides decision support in difficult treatment scenarios. *Disadvantages:* invasive; requires calibration with built-in blood glucose meter; requires a change of the sensor after a few days. Reliability: NA* *Cost:* US $1,524 for the receiver and transmitter and US $64.20 for a sensor; daily cost of US $7.50. *Availability:* a few European countries, South America, India
HG1-c (C8 Medisensors)	*Target site:* skin *Technology used:* Raman spectroscopy *Advantages:* compact, wearable and light-weight; non-invasive glucose monitoring; CE-approved; small and water-resistant glucose sensor; glucose measurement every 5 min; precision comparable to blood glucose meters; transmission of CGM data continuously to a smartphone; smartphone can view 3 h of instantaneous readings and the previous four months’ readings for a retrospective view; customized personal alerts for high and low glucose levels; no requirement for constant recalibration; cost-effective compared to fingerstick tests based on estimates for four years with three fingerstick tests per day. *Disadvantages:* approved for investigational use in the US. *Reliability:* 53% in A and 92% in A + B zones of EGA [62]. *Cost:* US $4,000 for CGMS. *Availability:* NA*
GlucoTrack™ (Integrity Applications Ltd.)	*Target site:* ear lobe skin *Technology used:* ultrasound, electromagnetic and heat capacity *Advantages:* compact and light-weight; large LCD screen; high precision due to the use of various non-invasive glucose monitoring techniques; alerts for hypo- and hyper-glycemia; multi-user support; easy calibration procedure with the calibration being valid for a month; USB and infra-red (IR) connectivity; data storage capacity; software for data analysis; readings unaffected by daily routine activities; good correlation with glucose meters and glucose analyzers; and high accuracy in clinical trials. *Disadvantages:* must be individually calibrated against invasive basal and post-prandial blood glucose references before use; requires tremendous improvements in the calibration procedure and algorithm for data processing. *Reliability:* 92% in A + B with 50% in the A zone of EGA. The MARD and median relative absolute difference were 29.9% and 19.9% [63]. *Cost:* US $1,926 for CGMS. *Availability:* NA *
OrSense NBM-200G (OrSense Ltd.)	*Target site:* fingertip skin *Technology used:* occlusion near infrared spectroscopy *Advantages:* portable; easy-to-use; measures glucose in less than a minute; data storage capacity, alerts for hypo- and hyper-glycemia; data analysis trend; integrated wireless telemetry; allows non-invasive glucose measurement together with hemoglobin and oxygen saturation; easy calibration procedure with no requirement for frequent calibrations; CGM for 24 h; good accuracy in clinical trials; precision comparable to glucose meters; and CE approved. *Disadvantages:* NM ** Reliability: 95.3% in the A + B and 4.7% in C + D zones of EGA. *Cost:* NA* Availability: NA *
Symphony^®^ (Echo Therapeutics, Inc.; previously Sontra Medical Corporation)	*Target site:* skin *Technology used:* Prelude® SkinPrep System *Advantages:* compact and light-weight; measures glucose every min; integrated wireless telemetry; alarm alerts for rapid changes in glucose levels; brief warm up period; no skin irritation; highly successful clinical trials; good correlation with glucose analyzers and glucose meters. *Disadvantages:* NM ** Reliability: The accuracy was determined in three study groups, *i.e.*, study I in diabetics, study II in patients undergoing cardiac surgery and study III in healthy volunteers [64]. There were 89.6%, 86.4% and 89.9% readings in zone A, and 9%, 13.6% and 10.1% readings in zone B of study I, II and III, respectively. *Cost:* NA * Availability: NA *

* NA: Not Available; ** NM: Not Mentioned.

## 2. Continuous Glucose Monitoring Systems

### 2.1. Dexcom SEVEN^®^ Plus

The Dexcom SEVEN*^®^* Plus (DSP) [65] was the previous generation of successful CGMS from Dexcom. It has a transmitter that is put on the body and a sensor that goes into the transdermal layer of the skin for measuring the glucose levels in the interstitial fluid. The sensor, comprising a miniaturized, small and round wire, has been approved by the United States Food and Drug Administration (FDA) and can be used for up to seven days. It attaches to the skin with its adhesive patch and can detect the glucose levels in hypoglycemia with excellent accuracy. The transmitter is water-resistant, has a built-in battery that lasts one year and is equipped with wireless capabilities to transfer the glucose sensing data to the handheld receiver within a five-foot range every 5 min. The receiver’s screen shows the trends and real-time glucose information. It can store up to 30 days of glucose trends along with a record of activities and events for more effective diabetic management. DSP comes with Dexcom*^®^* Data Manager 3 software, which provides better insight into ways of improving glycemic control.

The accuracy and safety of the DSP and FreeStyle Navigator^®^ (FN) (Abbott Diabetes Care, Alameda, CA, USA) was evaluated with the YSI blood glucose analyzer [57]. DSP was better in the hypoglycemia region (YSI value < 80 mg/dL), as its mean absolute relative difference (MARD) (21.5%) was lower than that of FN (29.8%). Moreover, it caused less skin reactions, *i.e.*, three *versus* 14 in comparison to FN. However, the MARD values of two CGMS were nearly the same *versus* the YSI analyzer, *i.e.*, 16.8% and 16.1% for DSP and FN, respectively.

A study was performed to compare DSP in type 1 diabetic patients on multiple daily injection (MDI) and continuous subcutaneous insulin infusion (CSII) therapy for six months [66]. Both study groups had similar changes in mean glucose and glucose variability at three and six months. However, CGMS use of at least six days/week demonstrated greater improvement in the time spent in the target glycemic range of 70–180 mg/dL in the CSII group. 

### 2.2. Dexcom G4^TM^ PLATINUM

Dexcom G4^TM^ PLATINUM (DG4P) [67] was the following highly improved wearable CGMS that was launched by Dexcom in 2012 after Dexcom SEVEN^®^ Plus (DSP). It measures glucose continuously every 5 min using a very thin sensor that has the same diameter as a human hair. The transmitter has advanced wireless capabilities that enable the transmission of readings to the receiver up to a distance of 6 m. DG4P is compact, thinner and around 30% lighter than DSP. Moreover, it has >20% higher accuracy in glucose measurements in comparison to DSP. The MARD of DG4P was 14%, which was lower than that of DSP, *i.e.*, 16% and comparable to that of blood glucose meters, *i.e.*, 10%–15%. As per the data shown on Dexcom’s website, 97% of the glucose measurements fall in the A and B zones of Clarke error grid analysis. The receiver has a color screen and a range of customizable alarms to alert for high and low glucose levels in addition to when the glucose levels are rising or falling quickly and when the receiver is out of wireless range from the transmitter. The default setting for the alarms and alerts for high and low glucose levels are above 11.1 mmol/L and below 4.4 mmol/L, respectively, which are customizable and can be switched off. However, there is an additional default alarm that cannot be turned off, *i.e.*, when the glucose level drops down to 3.1 mmol/L. DG4P requires calibration by blood glucose testing once every 12 h. It also requires the change of the sensor whenever it is indicated on the receiver’s screen. However, usually the sensor can be used for up to seven days, while the battery of the transmitter lasts about six months after which it needs to be replaced. DG4P comes with the Dexcom STUDIO^TM^ data management software, which enables simple navigation and illustrative visuals for the quick and easy identification of glucose patterns.

The performance of DG4P and DSP was compared with that of the YSI blood glucose analyzer in a seven-day multicenter pivotal study [58]. DG4P performed significantly better than DSP, as it had a lower aggregate MARD of 13% in comparison to 16% for DSP. Ninety percent of DG4P sensors had an individual MARD of ≤20% in comparison to 76% for DSP. DG4P had a mean absolute difference of 11 mg/dL for hypoglycemia (YSI < 70 mg/dL) compared with 16 mg/dL for DSP. 82% of DG4P values and 76% of DSP values were within 20% of YSI values (for YSI > 80 mg/dL).

### 2.3. Guardian^®^ REAL-Time

The Guardian*^®^* REAL-Time CGMS (Medtronic, Minneapolis, MN, USA) [62,68,69] comprises a standalone receiver and a wireless transmitter attached to a sensor, which goes into the transdermal layer of skin to continuously measure the glucose levels in the interstitial fluid every 10 s. The average glucose reading is then transmitted to the monitor every 5 min. The receiver displays the glucose trends and enables the users to set up alarms for low and high glucose levels. It has predictive alerts to notify the user up to 30 min before they are predicted to reach a personal low or high glucose level. The trend arrow also informs the user if the glucose levels are rising or falling quickly. However, the sensor, which lasts up to six days, costs around US $78. The Minimed Paradigm*^®^* REAL-time Revel^TM^ system is the newly developed second generation product that combines an insulin pump with the CGMS.

The effect of Guardian*^®^* REAL-Time CGMS on glycemic control was evaluated [48]. It was observed that the CGMS provided real-time glucose readings, highly useful information about glucose excursions and trend information on changing glucose values. This led to a reduction in the HbA1c level from the baseline *versus* control at one month (0.6 ± 0.8 *vs.* 0.2 ± 0.8%, *p* = 0.008) and three months (1.0 ± 1.1 *vs.* 0.4 ± 1.0%, *p* = 0.003). There was no significant reduction in the average total insulin dose per day at three months. However, almost all patients, 82% at one month and 95% at three months, were reported to make insulin, dietary and/or lifestyle adjustments using the real-time glucose values and information provided by the CGMS. Another study also demonstrated that the use of Guardian*^®^* REAL-Time CGMS led to significantly improved HbA1c values just within three months in type 1 diabetics, despite intensive insulin therapy [70]. This enables patients to improve their glycemic control by adjusting their insulin doses, food intake and physical activity. Similarly, it was recently demonstrated that Guardian*^®^* REAL-Time CGMS was safe and useful in critically ill children with 72% and 27.6% values in zones A and B, respectively, of Clarke (EGA) and a mean absolute relative deviation of 17.6% [59].

A study demonstrated that the mean absolute relative error between blood glucose meter and Guardian*^®^* REAL-Time CGMS readings was 21.3% [71]. The hypoglycemia alert distinguished blood glucose values ≤70 mg/dL with sensitivity, specificity and false alerts of 67%, 90% and 47%, respectively. While the hyperglycemia alert detected blood glucose values ≥250 mg/dL with sensitivity, specificity and false alerts of 63%, 97% and 19%, respectively. Therefore, Guardian*^®^* was found to be reasonable accurate in CGM. The subjects’ responses to hypoglycemia alerts led to a significant decrease in the duration of hypoglycemic excursions. On the other hand, the overtreatment of hypoglycemia might have led to a marginally significant increase in hyperglycemic excursions, but with a significant decrease in their duration.

The accuracy of Guardian*^®^* REAL-Time CGMS was further evaluated in intensive care unit (ICU) patients [72]. It was more accurate in euglycemia, with the mean absolute deviation (MAD) and MARD being 28.3 mg/dL and 17.4%, respectively. The linear regression coefficient of CGMS on fingerstick blood glucose was 0.834 (*p* < 0.001). Therefore, it was not accurate enough for therapeutic decisions in the ICU, especially in diabetic ketoacidosis patients. Moreover, there were technical difficulties, such as the requirement for adequate time for electrode wetting and calibration of electrodes.

The Minimed Paradigm*^®^* REAL-time Revel^TM^ system, launched in 2006, stores highly useful information about glucose, insulin and meals, which is provided to the healthcare professional by CareLink^TM^ Personal therapy Management software. It has the distinct ability of observing and reacting quickly to glucose fluctuations using the Bolus^®^ Wizard calculator feature that is available in the insulin pump [73]. It allows the users and healthcare professionals to modify the insulin therapy for better glycemic control. 

### 2.4. FreeStyle Navigator^®^

**Figure 3:**
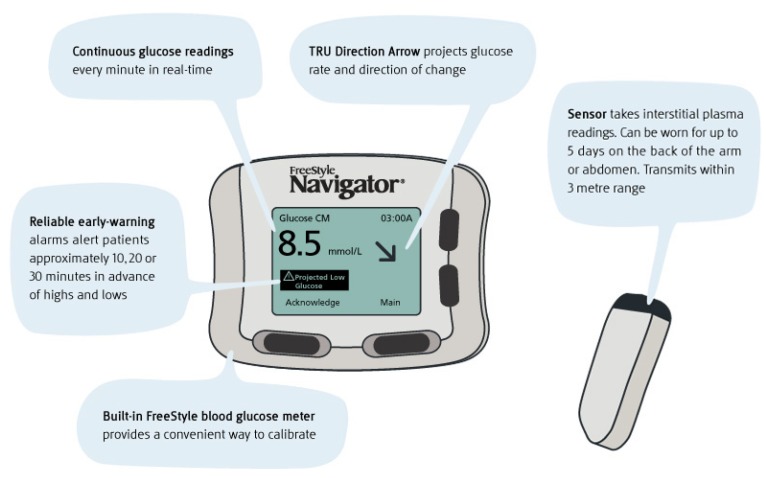
Visual interpretation of the indicators on CGMS.

FreeStyle Navigator^®^ (FN) [74] is a CGMS from Abbott that measures glucose level every minute. It consists of a sensor, a transmitter that is attached on the body and a handheld receiver. The sensor is inserted into the body, usually in the abdomen or the back of the upper arm, using an insertion device. It employs a solid adhesive pad for providing a safe and solid base to attach the wireless transmitter, which can transmit the glucose measurement data to the receiver up to a distance of 3 m. However, the receiver in FreeStyle Navigator^®^ is a stand-alone unit with integrated blood glucose testing. The receiver’s screen shows the current glucose level, glucose trend and the continuous glucose measurements. The transmitter is waterproof (up to 45 min in a depth of 1 m), light (13 g), compact (5.2 × 3.1 × 1.1 cm) and employs a battery that lasts for about 30 days. The handheld receiver is of the size of a cellphone (6.3 × 8.2 × 2.2 cm), weighs 99 g and employs 2 AAA batteries that last up to 60 days. The built-in FreeStyle blood glucose meter provides convenient calibration using the test strips used in Abbott’s FreeStyle Lite blood glucose meter. The receiver provides reliable early-warning alarms that alert patients to the high and low glucose levels in less than 30 min in advance (Figure 3). 

The performance of FN was inferior to DSP, due to lower accuracy in the hypoglycemia region and significantly more skin reactions, but the MARD values of both CGMS devices were nearly the same as the YSI analyzer [66]. Moreover, the first generation of FN requires a 10 h warm up period in order to avoid inaccurate glucose readings, which are caused by sensor insertion and wound-healing. However, this was improved in the second generation of FN, where the warm up period was reduced to 1 h. A study demonstrated 93.7% readings in the clinically accurate zone of EGA, while 3.6% and 2.8% readings were in the benign error and clinical error regions, respectively [60]. The clinical accuracy of the FN in the first 10 h and subsequent hours was 92.6% and 94.2%, respectively. Another study determined that FN has the MARD and median absolute relative difference of 12.8% and 9.3%, respectively [61]. The readings in the clinically accurate A and benign error B zones of EGA were 81.7% and 6.7%, respectively. The measurements of FN were consistent and accurate in comparison to the venous measurements made using the YSI blood glucose analyzer for five days. It was found that 82.5% and 80.9% of FN readings were in the A zone of EGA on the first and fifth day, respectively.

### 2.5. FreeStyle Navigator II

**Figure 4:**
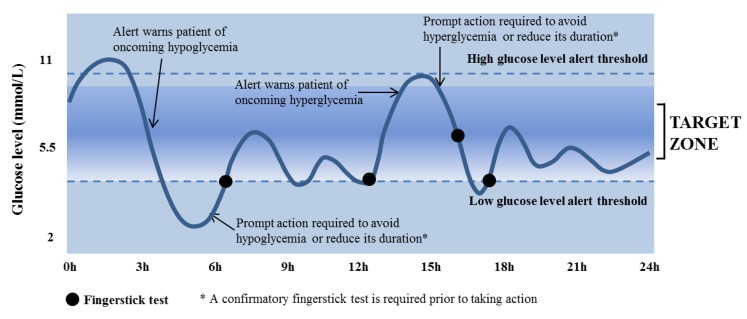
Visualization and interpretation of the continuous glucose monitoring (CGM) curve.

Abbott has recently developed FreeStyle Navigator II [75], which is an advanced version packed with more features that is small, lightweight and comfortable to wear. It has a capsuled battery in the transmitter and a capsuled reloadable battery cell in the receiver that lasts for one and two years, respectively. Although the sensor is the same as employed in FreeStyle Navigator^®^, the transmitter and receiver are smaller. It has increased the number of sounds for the alarms. The sensor pricing is the same, but the starting kit is 25% more expensive. It provides a continuous update of glucose levels every min, blood glucose averages of the last 10 min and the low and high blood glucose levels. The sensor can be used for continuous glucose monitoring (CGM) for up to five days. The receiver is easy to use with an illuminated color display. It provides glucose trend along with its direction and early warning alarms if there is a risk of too low or too high glucose levels. The schematic of the CGM curve along with its interpretation is shown in Figure 4. The remarkable feature of FreeStyle Navigator II is its non-interference with the everyday activities of the diabetic, thereby providing much desired flexibility. It works during showering, swimming, exercising or traveling and can transmit glucose sensing data to the receiver at an extended range of 30 m. In comparison to other CGMS that are intended and can only be used by adults, FreeStyle Navigator II can also be employed for children above six years of age and adolescents under the supervision of an adult. It has a built-in FreeStyle blood glucose meter that uses the FreeStyle Lite blood glucose test strips, ZipWik, which have a unique design for accurate and precise glucose measurements in 4 s. It is also appropriate for evaluating blood glucose fluctuations in the short term and additionally provides decision support in difficult treatment scenarios.

### 2.6. HG1-c

C8 MediSensors (San Jose, California, USA) has recently developed a Conformité Européenne (CE)-approved compact non-invasive CGM device named HG1-c [36]. It is based on the principle of Raman spectroscopy, where a painless pulse of monochromatic light is transmitted into the skin, and the scattered light is detected for the determination of glucose levels. It employs proprietary sophisticated regression analysis techniques for the highly specific detection of the Raman signature of glucose, which can be discriminated from the Raman spectra of other compounds in the body. However, it has been approved for investigational use in the US. The adjustable band containing the glucose sensor is designed to be worn on the abdomen. The sensor is small, water-resistant, performs glucose measurement every 5 min and has precision comparable to the commercially-available invasive blood glucose meters. The CGM data is continuously transmitted to a smartphone, which can view 3 h of instantaneous readings and the previous readings for four months for a retrospective view. The users can set up the alerts for high and low glucose levels. The main feature of the device is that it does not require constant recalibration, which is essential for invasive CGMS. Moreover, it has significantly reduced the glucose monitoring cost, which has been claimed to be less than three fingerstick tests per day over four years. C8 MediSensors has raised US $24 million, with investors, such as GE Healthcare, Venture Lending, Leasing VI, Inc. and an investor from Dubai, for its planned product introduction in Europe in 2013. However, the company was suddenly closed in January, 2013, and the company’s website was taken offline immediately. From the Internet blogs, it seems that the company went into financial difficulties, and the device performance was inadequate as per the established CGMS performance standards. Based on the study published in 2009 [76], the device had 53% and 92% readings in the Clarke A and A + B zone, respectively, whereas the existing CGMS standards state 73% and 97% readings to be in the Clarke A and A + B zones, respectively [62].

At the Consumer Electronics Show, 2013, iHealth Lab Inc., California, USA, recently announced a similar iPhone operating system (iOS)-enabled smartphone-based Wireless Smart Gluco-Monitoring System [77] that can store up to 500 blood glucose test results wirelessly, track the trends and set up medication alerts. It will be commercially introduced in the second half of 2013 after getting FDA approval. 

### 2.7. GlucoTrack ^TM^

GlucoTrack^TM^, developed by Integrity Applications Ltd., Ashkelon, Israel, is a compact, lightweight and real-time non-invasive CGM device, which determines blood glucose concentration using three NGM techniques, *i.e.*, ultrasonic, electromagnetic and heat capacity [78]. It was approved by CE very recently. The use of various techniques minimizes the effect of interference, thereby providing higher precision and accuracy. It measures glucose concentration in the earlobe using a personal ear clip (PEC) equipped with sensors and calibration electronics. The use of the earlobe, which has abundant blood supply and is easily accessible, does not affect the routine activities of the subject. It requires an individual calibration against invasive basal and post-prandial blood glucose references that remains valid for a month before it can be used for CGM. The device has a rechargeable battery, a large LCD screen, multi-user support, software for data processing and analysis, USB and infra-red (IR) connectivity, alerts for hypo- and hyper-glycemia and can store up to 1,000 readings per user. However, it has not been commercialized, as the company intends to further improve its analytical performance by developing a better calibration procedure and an improved algorithm for data processing. The clinical trials to evaluate the correlation of its readings with those from commercial glucose meter and glucose analyzer stated 92% of its readings to be in the clinically acceptable A and B zones of Clarke EGA with 50% in the A zone [63]. The MARD and median relative absolute difference were 29.9% and 19.9%, respectively. Therefore, further research efforts were desired to significantly improve the accuracy of GlucoTrack^TM^. 

### 2.8. OrSense NBM-200G

OrSense NBM-200G, developed by OrSense Ltd., Petah-Tikya, Israel, is a portable and easy-to-use NGM technique-based CGM device that measures glucose, hemoglobin and oxygen saturation with a very high sensitivity in less than a minute [36]. It is a CE-approved device based on the principle of near infrared (NIR) occlusion spectroscopy, which detects the red NIR optical signal of blood due to changes in the glucose concentration in the blood vessels of the finger. The signal-to-noise ratio is improved by the temporary cessation of blood flow to the finger in occlusion, which enhances the red NIR signal. It is equipped with alarm alerts, trend data analysis, integrated wireless telemetry, an easy to read display and can store up to 500 readings. It measures glucose continuously for up to 24 h without any requirement for frequent calibrations and is completely safe for patients, without any risk of contamination. The information available on the company’s website states its accuracy to be comparable to blood glucose meters based on the tests performed in over 400 subjects. It identified the glucose trends and detected the hypo- and hyper-glycemia events. The clinical trial conducted at the Sheba Medical Center, Israel, and in an outpatient clinic stated that 95.3% of its readings were in the Clarke error-grid’s A and B zones, 4.7% of points in the C and D zones and no points in the E zone. Israel Health Care Ventures and STAR Ventures were the investors in OrSense’s CGM technology, which received the Frost and Sullivan’s Technology Innovation award in 2006. However, the device was not commercialized and is only being used for investigation and market awareness.

### 2.9. Symphony^®^

Symphony^®^ was the non-invasive CGM device that was developed by Sontra Medical Corporation (presently Echo Therapeutics, Inc., Philadelphia, PA, USA). It employs a standard glucose sensor to measure transdermal glucose levels [36]. It involves a special transdermal permeation system and a brief warm-up period before the CGM every minute on the permeated site. The data is transmitted wirelessly to a remote monitor, which is equipped with alarm alerts if the glucose levels go outside the normal physiological range. The device does not cause any skin irritation, has no safety concerns and demonstrated good clinical performance in the clinical trials conducted in 2011. The glucose levels determined by Symphony^® ^ correlated well with the blood glucose levels determined by the YSI 2300 STAT Plus glucose analyzer and commercial glucose meters. The results reported in January 2012, stated that 96.9% of values were in the A and B zones of the Clarke EGA for 20 volunteers. The accuracy of the device was determined in three study groups, *i.e.*, study I in diabetics, study II in patients undergoing cardiac surgery and study III in healthy volunteers [64]. There were 89.6%, 86.4% and 89.9% readings in zone A, and 9%, 13.6% and 10.1% readings in zone B of study I, II and III, respectively.

## 3. Challenges

The key challenges for the development of next-generation CGMS for diabetic management are the decrease in the operational cost, development of prospective NGM techniques for precise and specific glucose detection, significant reduction or obviation of calibration and warm up periods, improvement of signal-to-noise ratio (SNR) and sensitivity, development of wearable CGMS, evaluation of analytical performance and reducing the time taken for glucose measurement. 

The analytical performance of CGMS needs to be critically evaluated in terms of the EGA [79,80] and International Organization for Standardization (ISO) Standards [81]. The glucose measurements performed by the CGMS should correlate well with those performed by the glucose analyzers and glucose meters. The MARD [82] should be below 15%, while 95% of the glucose measurements should fall within ±15 mg/dL of a reference measurement of glucose concentration <75 mg/dL and within ±20% at glucose concentrations >75 mg/dL. 

It has been demonstrated that CGMS cannot replace blood glucose meters, due to their lack of performance as per the established glucose monitoring standards. A study showed that only 39% of CGMS readings satisfied ADA precision criteria to be within ±10%, while 19% of readings satisfied ADA precision criteria of accuracy to be within ±5% [83]. The CGMS readings in zone A and zone A + B of EGA were 77% and 98.7%, respectively, while the CGMS sensitivity and specificity to detect hypoglycemia were 33% and 96%, respectively. However, CGMS was found to provide more extensive information pertaining to the glucose profile than the blood glucose meters, which would be highly useful to healthcare professionals for effective diabetes management.

The SNR and sensitivity of CGMS, especially those based on NGM techniques, can be improved by using novel transducers and methods for the parallel monitoring of multiple parameters. This is employed in the GlucoTrack^TM^ NGM device, which employs ultrasound, conductivity and heat capacity for blood glucose determination. The SNR can be further improved by using digital filters and data treatment methods, such as ridge regression, artificial neural networks, principal component analysis and partial least squares. The development of prospective NGM technique-based CGMS is still a major challenge. However, it will be a boon to diabetics and healthcare professionals for highly effective diabetic management, as they can alleviate the pain, discomfort, suffering and issues related to biocompatibility. The recent development of NGM technique-based wearable CGMS, *i.e.*, HG1-c by C8 MediSensors, was a remarkable achievement in this regard.

There is a need to develop procedures for determining the blood glucose levels from the glucose concentration in other physiological fluids, such as interstitial fluid. Although several groups have developed Dynamic Concentration Correction (DCC)-based calibration procedures [84] for highly precise blood glucose determination, they still need to be validated by clinical studies. On the other hand, the “lag” between blood and tissue glucose also depends on the site of measurement, on individual patient characteristics and on whether the glucose is increasing or decreasing. Therefore, dynamic correlation methods may not necessarily be as useful. There is a physiological time lag for the glucose to pass from the blood to the glucose measurement site. Moreover, the glucose measurement procedure also induces time lag. The blood glucose, which is accepted as the standard, because of its ease of measurement, might not actually be the most relevant physiologically important parameter, as capillary blood might be better. 

Though far from perfect, the invasive technique-based CGMS have much better analytical performance than the NGM technique-based CGMS, as they employ the physiological glucose sample. Many NGM techniques have much higher response times for glucose determination in comparison to glucose meters, but the most recent development of a portable NGM device measuring glucose in just 1 s [85] is a noteworthy achievement. The ongoing research efforts will provide the essential tools to tackle the issues of the inherent lack of specificity, weak signal and interference from the absorption and scattering of other tissue components, which need to be addressed for certain NGM technologies. 

The recent developments in NGM by researchers at Massachusetts Institute of Technology (MIT, Cambridge, MA, USA) and University of Missouri-St. Louis (UMSL, St. Louis County, MI, USA) show a significant promise for the development of CGMS. The researchers at MIT developed a Raman spectroscopy-based NGM device [86] that measures glucose in the interstitial fluid by scanning the finger or arm with NIR light having a penetrative depth of 0.5 mm below the skin. They also developed an advanced algorithm to determine the blood glucose level from the glucose concentration in interstitial fluid. Additionally, a DCC-based calibration procedure was also devised for precise blood glucose monitoring, taking into account the rate of glucose diffusion from the blood into the interstitial fluid [82]. On the other hand, the researchers at UMSL developed a portable NGM detector [84] to determine the blood glucose levels in the capillaries of the finger with high precision in just one second, which is the most rapid response time for glucose monitoring. The procedure involved the shining of NIR light on the finger, detection of the light transmitted through the finger and providing the output signal to a processor that determines the glucose concentration. The technology has been licensed to St. Louis Medical Devices situated at the UMSL startup company incubator, which will conduct the clinical trials and get the regulatory approvals for the device before market entry. Despite all the challenges and technology concerns, there have been significant advances in the field of CGMS.

It has been established by several studies that the use of CGMS improve clinical outcomes in patients with diabetic mellitus [40,42,87,88,89,90,91,92]. These include improvements in HbA1c, glucose variability, time spent in hypoglycemia and hyperglycemia, quality of life and diabetes management. The American Association of Clinical Endocrinologists recommends the use of CGMS in type 1 diabetes mellitus patients who have frequent hypoglycemia, hypoglycemic unawareness or an HbA1c level above target, those requiring a reduction in HbA1c without increased hypoglycemia and during preconception and pregnancy [46]. CGMS provides real-time glucose values that allow for immediate therapeutic adjustments. In another study, the use of CGMS was recommend in children, adolescent and adult outpatients [87]. However, it was not recommended in adult hospital settings. Moreover, due to paucity of data, the use of CGMS in children (<8 year) could not be evaluated. 

The benefit of using CGMS is clearly evident from sensor augment pump (SAP) therapy, which involves the combining of CGMS with the insulin pump [93,94,95,96,97,98,99]. It demonstrated a significant reduction in HbA1c level, improvement of glycemic control and critically reduced severe hypoglycemic and hyperglycemic events in patients with type 1 diabetes mellitus in a rapid, sustainable and safe manner. The type 1 diabetics, who had a high HbA1c level and were older at diagnosis and randomization, experienced the most benefit from SAP therapy [94]. The subjects treated by SAP therapy for 12 months reported significantly less hospitalization, increased treatment satisfaction and reduced fear of hypoglycemia [95]. It was further demonstrated that the beneficial effects of SAP therapy persist, even after 36 months of its start [96]. The HbA1c level decreased from 8.7% to 7.3% from the time when the SAP therapy was started to the end of 36 months (*p* < 0.0001). A study demonstrated that the initiation of CGMS before subcutaneous insulin infusion increases the CGMS frequency use in type 1 diabetes mellitus patients and significantly reduces the time spent in hypoglycemia [89].

Moreover, the use of CGMS as an adjunct therapy to self-monitoring of blood glucose, as shown by the Juvenile Diabetes Research Foundation (JRDF) CGM trial [100], demonstrated that the frequent use of CGMS in children and adults with type 1 diabetes mellitus for six months was associated with significantly greater HbA1c reductions and less severe hypoglycemic events [46,47,48,49,50]. The trial further showed that young patients using CGMS for 6–7 days/week reduced their HbA1c level by a mean of 0.8% and were able to maintain this improvement for a year. It was also observed that insulin pump and CGMS users achieved an HbA1c level of <7% significantly more often than insulin pump and non-CGMS users. Therefore, CGMS is useful as an adjunct device along with blood glucose meters to investigate the glycemic patterns of diabetic patients. CGMS is also useful for guiding treatment adjustment. Studies have further suggested that physical activity (PA) interventions using CGMS feedback for type 2 diabetics may improve PA levels and reduce the risk factors for diabetic complications [49]. The moderate activity minutes increased significantly (*p* < 0.05), while HbA1c level and body mass index decreased significantly (*p* < 0.05). CGMS would be of immense utility in exercise and health science, as it will facilitate the improvement of a glucoregulatory exercise program and the development of much better evidence-based physical activity guidelines for glycemic control [101]. Additionally, CGMS was very useful in the management of gestational and pre-existing diabetes in pregnancy, where it provided additional information in 62% of pregnant women that altered clinical management decisions [102]. It enabled the detection of postprandial hyperglycemia and overnight hypoglycemia.

Researchers have also demonstrated the ability to detect incorrect readings from the raw data and information provided by CGMS using the Gaussian support vector machine classifier [103]. The classifier was trained on monitoring CGMS electrical signal and estimating glucose after multiple runs of many-fold cross-validation. The average specificity and sensitivity of CGMS after 10 runs of 10-fold cross-validation were 92.74% and 75.49%, respectively, while the average correct rate was 91.67%. The classifier detected the time intervals when CGMS glucose readings were incorrect, thereby enabling the detection of missed hypoglycemic episodes. Hypoglycemia has been regarded as the main barrier to glycemic control [104]. A separate study further demonstrated the need for both healthcare professionals and patients to have the skills required to interpret CGMS data [105]. 

## 4. Conclusions and Future Trends

Diabetes has become a global epidemic during the last two decades. It is increasing at an alarming pace of 7.8 million new diabetics each year and is taking an unsustainable economic toll, amounting to 11.6% of the total healthcare expenditure [1]. The ongoing research efforts to find the cure for diabetes by developing an artificial pancreas or by islet cell transplantation will take a lot of time based on the challenges involved and only a very limited scope for success. The current use of intensive insulin treatment is inadequate, as it leads to a marked increase in episodes of severe hypoglycemia. Moreover, insulin treatment is not expected to dramatically reduce the formation of Amadori products, which have a major impact on secondary health problems. Therefore, more frequent glucose monitoring is the only way to effectively manage diabetes by sustaining the physiological blood glucose level. 

CGMS enables diabetics to continuously monitor their glucose level, which has been shown to significantly lower the HbA1c levels in diabetics and, thereafter, sustain it for a long period of time. A five-week pilot study in the initial phases [50] demonstrated the decrease in HbA1c from 9.9% (SD = 1.1%) at baseline to 8.8% (SD = 1.0%) five weeks after baseline. However, there was no change in the daily insulin usage. CGMS performed accurately, with a median correlation and mean absolute difference of 0.92% and 19.1% (SD = 9.0%), respectively. It has been shown that CGMS detects the abnormal patterns of glycemia many folds better than the conventional technology in pediatric type 1 diabetics and further enables a significant decrease in HbA1c from 8.4 ± 1.5% at baseline to 8.4 ± 1.3% at three months [54]. The reduction of the HbA1c level is critically important, because it has been shown that even a 0.5% reduction in the HbA1c level can reduce the risk of retinopathy by 25%. The accuracy of CGMS is still a key issue for children and adolescents, who have increased variability in blood glucose. The significantly higher costs of CGMS [11], presently around 78 USD for six days, strictly limit its routine use by diabetics. Initially, the CGMS were not reimbursed by insurance companies in many countries. However, many healthcare providers now reimburse CGMS for specific target groups, where it is highly required [46]. However, the inadequate reimbursement of clinicians’ time is a barrier to the adoption of CGMS [106]. It has also been shown by several studies and trials that the use of CGMS may not significantly reduce the number of hypoglycemic episodes, but it reduces the time spent in hypoglycemia. Similarly, other studies state that CGMS are not more useful than the fingerstick-based blood glucose meters [107,108]. Moreover, the variations in glucose levels in the interstitial tissue, as determined by CGMS, do not coincide perfectly with those in blood [109]. On the other hand, other studies have shown the low efficacy of CGMS in detecting unrecognized hypoglycemia in type 1 diabetic mellitus patients, where CGMS presented low sensitivity (79.1%) to detecting hypoglycemia *versus* hyperglycemia (96.8%) [53]. The CGMS studies in children with type 1 diabetes mellitus have obtained conflicting results. A study demonstrated that CGMS did not lead to significant reduction in HbA1c levels in comparison to the control groups, increased the number of insulin dose changes per patient per month and was not better than the blood glucose meter [108]. Another study also concluded that there is insufficient evidence to support that CGMS is superior to blood glucose meters in HbA1c reduction [110]. However, it also stated that there was some indication that CGMS leads to improved detection of asymptomatic nocturnal hypoglycemia. However, a more recent study showed that CGMS significantly improves the HbA1c levels apart from reducing the time spent in the hypoglycemic range, when it is used as part of SAP therapy [111]. These effects of CGMS are enhanced when it is used daily along with insulin pump therapy.

Most of the commercially-available CGMS are based on invasive techniques and employ a complex procedure, where the diabetic has to be trained and educated to use it. They do not obviate the fingerstick blood tests by glucose meters, as the CGMS still needs to be calibrated many times by blood glucose meters. Moreover, there is a need to change the invasive sensor of CGMS after every few days. In addition, there is associated patient discomfort and skin irritation. Therefore, there is a critical need for cost-effective NGM technique-based CGMS that can alleviate the pain and suffering associated with glucose monitoring, which will motivate diabetics to use the CGMS to sustain euglycemia. The development of HG1-c by C8 MediSensors was a major step forward in this direction for the development of next-generation CGMS. There is a need for more intensive research efforts in the future to develop robust, highly precise and specific CGMS, which will employ advanced algorithms to take into account the time lag between the concentration of glucose in the blood and the interstitial fluid [112]. The number of calibrations required for CGMS also need to be obviated or reduced. Moreover, it should be noted that CGMS is intended to be used as a complimentary device that cannot replace blood glucose testing, as per the established healthcare guidelines. 

CGMS still requires tremendous improvements in order to address the challenges of cost-effectiveness, the use of NGM technology; rapid response, elimination of interference, higher precision, improved calibration, increased comfort and patient safety and significantly improved software and device features. However, these developments require substantial and continuous funding, which can only be afforded by industrial giants, such as Abbott, Dexcom, Roche and Medtronic. 

On the other hand, the widely-used blood glucose meters require only less than 1 µL of blood, which is obtained with a 32 gauge lancet that is not so painful and does not necessarily require the finger as the sampling site. Moreover, the test strips can also be made very reproducibly, which obviates the need for calibration. The existing glucose meters are highly compact, take only a few seconds for glucose measurement, contain internal memory for storing many glucose readings and are additionally provided with diabetes management software. However, the test strips are still expensive (often US $1 each), but the prices are expected to come down. Similarly, there have been continuous improvements in blood glucose monitoring and development of novel chemistries for the preparation of enzyme-bound strips [113,114,115,116]. However, the blood glucose monitoring devices have already reached the advanced stage with adequate analytical performance, cost-effectiveness, convenience, software-based data analysis and management and sophisticated device features. Therefore, there is no critical requirement for further technology improvement. 

Undoubtedly, CGMS will be highly useful for effective diabetic management and will enable diabetics to have a healthy lifestyle, but the biocompatibility issues for indwelling sensors is still a major problem that has only partly been solved. The current trend has shifted towards NGM-based CGMS, which have an additional benefit of employing a smartphone as the receiver for CGMS, which will have extensive applications in the near future in the fields of mobile healthcare and personalized medicine. However, the precision, robustness, stability and analytical performance of NGM techniques still require considerable improvements. There is an exclusive need for integrated and advanced diabetic management software, such as GluCoMo [117]. Based on the extensive ongoing research efforts and the needs of the continuously expanding diabetic community, the next decade will certainly witness the development of several truly innovative CGMS for effective diabetic management. 

The ongoing studies will further demonstrate the contribution of using CGMS towards the significant retardation of diabetes-associated sequelae related to glycemic and metabolic memory and secondary disease types. Here, a significant reduction of costs in a highly prevalent disease, such as diabetes, will testify to beneficial changes in healthcare management considering the long-term effects. CGMS will be highly useful in the ICU for the management of malignancies by carbohydrate-reduced diets. Abnormal blood glucose levels are quite common during critical illness and are associated with bad clinical outcomes [118]. Nevertheless, a large number of studies have been unable to prove the benefit of glycemic control, due to the insufficiencies of measuring glucose and insulin infusion therapy. Thus, there is a strong need for a closed-loop system incorporating CGMS and the insulin pump [119]. Recently, Inoue and colleagues [120] reported the lack of joint consensus from individual centers’ experiences. Moreover, there is a need to differentially evaluate the arterial blood glucose measurements from the capillary blood glucose meter results. The effect of variation in environmental and physiological factors on glucose measurements also needs to be studied. All these clinical studies will clearly illustrate the benefits of using CGMS, which will lend considerable support for the development of the next generation of CGMS.
